# The Northeast Atlantic is running out of excess carbonate in the horizon of cold-water corals communities

**DOI:** 10.1038/s41598-020-71793-2

**Published:** 2020-09-07

**Authors:** Marcos Fontela, Fiz F. Pérez, Lidia I. Carracedo, Xosé A. Padín, Antón Velo, Maribel I. García-Ibañez, Pascale Lherminier

**Affiliations:** 1 Instituto de Investigaciones Marinas, IIM-CSIC, 36208 Vigo, Spain; 2 Centre of Marine Sciences (CCMAR), University of Algarve, 8005-139 Faro, Portugal; 3 Ifremer, Univ. Brest, CNRS, IRD, Laboratoire d’Océanographie Physique et Spatiale (LOPS), IUEM, 29280 Plouzané, France; 4 Centre for Ocean and Atmospheric Sciences, School of Environmental Sciences, University of East Anglia, Norwich, UK

**Keywords:** Ocean sciences, Marine chemistry

## Abstract

The oceanic uptake of atmospheric carbon dioxide (CO_2_) emitted by human activities alters the seawater carbonate system. Here, the chemical status of the Northeast Atlantic is examined by means of a high-quality database of carbon variables based on the GO-SHIP A25 section (1997–2018). The increase of atmospheric CO_2_ leads to an increase in ocean anthropogenic carbon (C_ant_) and a decrease in carbonate that is unequivocal in the upper and mid-layers (0–2,500 m depth). In the mid-layer, the carbonate content in the Northeast Atlantic is maintained by the interplay between the northward spreading of recently conveyed Mediterranean Water with excess of carbonate and the arrival of subpolar-origin waters close to carbonate undersaturation. In this study we show a progression to undersaturation with respect to aragonite that could compromise the conservation of the habitats and ecosystem services developed by benthic marine calcifiers inhabiting that depth-range, such as the cold-water corals (CWC) communities. For each additional ppm in atmospheric pCO_2_ the waters surrounding CWC communities lose carbonate at a rate of − 0.17 ± 0.02 μmol kg^−1^ ppm^−1^. The accomplishment of global climate policies to limit global warming below 1.5–2 ℃ will avoid the exhaustion of excess carbonate in the Northeast Atlantic.

## Introduction

The uptake of anthropogenic CO_2_ by the ocean (C_ant_) creates a series of chemical changes known as ocean acidification^1^. The North Atlantic stores the largest amount of C_ant_ in the ocean^2,3^, with the Atlantic Meridional Overturning Circulation (AMOC) conveying and exporting acidified C_ant_-loaded waters to the deep ocean^4^. At basin-scale, North Atlantic Ocean acidification is a well-known process^5^, usually reported with pH decrease rates of ~ 1–2 × 10^–3^ pH units yr^−1^^6–9^. The reduction in the concentration of carbonate ions ([CO_3_^2−^]) is also a consequence of ocean acidification^10^, usually expressed as the change in the calcium carbonate (CaCO_3_) saturation state. The availability of carbonate connects the chemistry of the seawater with the biological activity, since carbonate is used by calcifying organisms to create the different forms of biogenic CaCO_3_: aragonite (corals, pteropods) or calcite (coccolithophores, foraminifera). The difference between the in situ [CO_3_^2−^] and the concentration at saturation is the excess of carbonate (_xc_[CO_3_^2−^])^10,11^. Positive values of _xc_[CO_3_^2−^] indicate supersaturated waters, while negative values indicate undersaturation and the tendency for the biogenic mineral to dissolve^12,13^. Ocean acidification decreases _xc_[CO_3_^2−^], compromising the fitness of marine calcifiers and even their survival when waters reach negative values of _xc_[CO_3_^2−^].

Cold-water corals (CWC) with biogenic CaCO_3_ skeletons made of aragonite^14^ are important ecosystem engineers of deep-sea habitats^15,16^. Ocean acidification is recognized as one of the most challenging threats that CWC will face with global change^17^. At global scale, large CWC reefs are more abundant in the North Atlantic, since the depth at which aragonite becomes susceptible of dissolution is deeper than elsewhere in the world ocean^4,18,19^. In the Northeast Atlantic, the relationship between reefs and hydrography is such that living CWC reefs are located in the potential density range 27.35 > σ_0_ > 27.65 kg m^−3^^20^. Recently, several Marine Protected Areas (MPA) have been proposed in European waters based on the presence of *Lophelia pertusa *sp. communities^21,22^. An assessment of the long-term viability of these hotspots of biodiversity and ecosystem services^14^ in the context of a changing ocean is therefore necessary.

**Figure 1:**
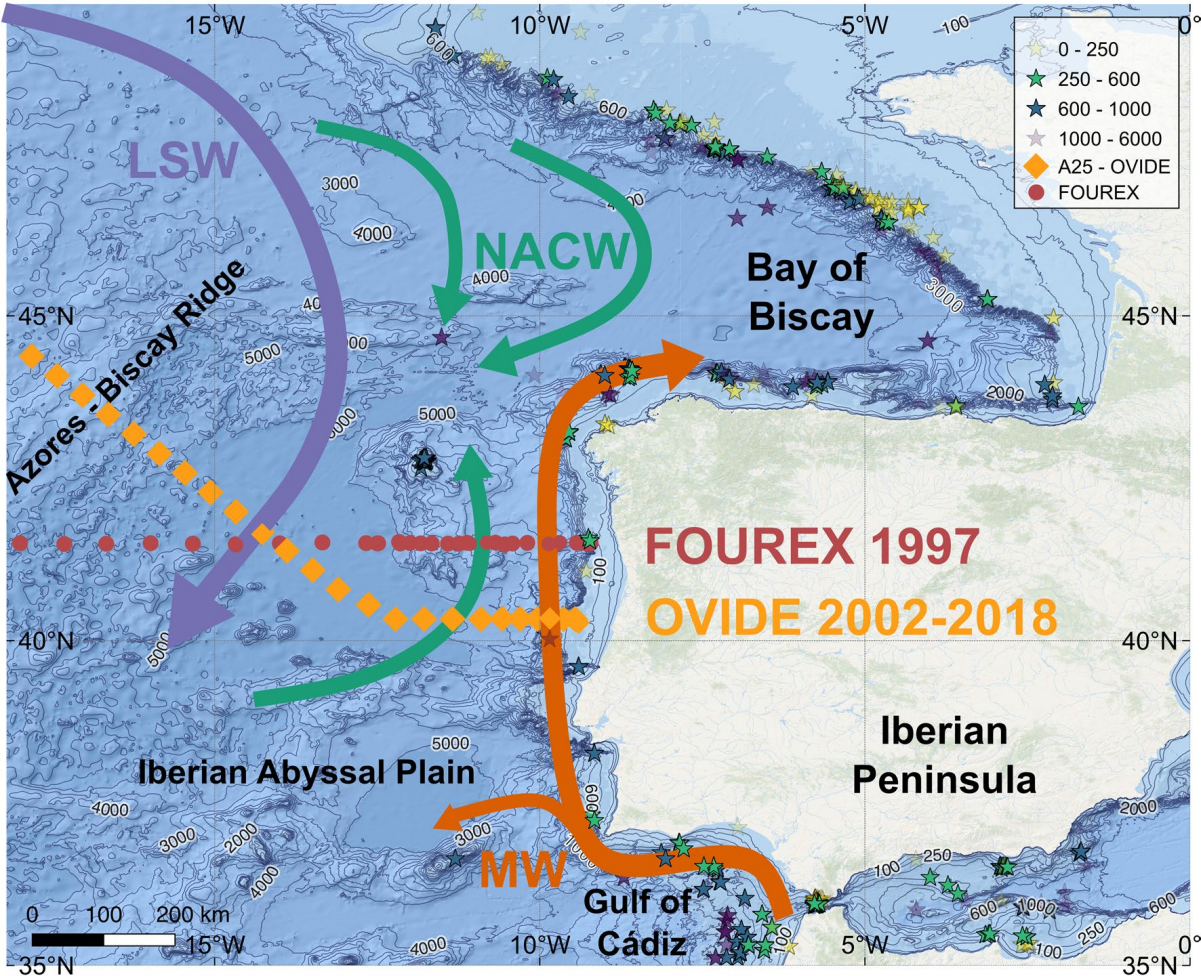
Map of the Northeast Atlantic zone of study close to the Iberian Peninsula. The location of the OVIDE (FOUREX) stations used in this study are represented with orange (red) diamonds (circles). The FOUREX section was carried out in 1997 and the OVIDE section has nine repeats, biennially from 2002 to 2018. The Azores-Biscay Ridge is the northern limit of the measurements used in this study. The locations with reported CWC^64^ communities where *Lophelia pertusa* is present are represented with stars. The stars are coloured according to the depth of the CWC location (see legend in Figure). A schematic diagram of the large-scale circulation of the main water masses is also shown: North Atlantic Central Water (NACW), Mediterranean Water (MW) and Labrador Sea Water (LSW), adapted from^32,65^.

This study relies on repeated marine chemical surveys across the A25 section of the Global Ocean Ship-Based Hydrographic Investigations Program (GO-SHIP, https://www.go-ship.org) to explore the implications of ocean acidification for marine calcifiers from a biogeochemical perspective (Fig. 1). The separation between natural and anthropogenic carbon allows the identification of ocean acidification trends driven by atmospheric CO_2_ increase. Although _xc_[CO_3_^2−^] decrease rates are scarce in the current literature of ocean acidification^23^, the use of this variable is particularly relevant to assess quantitatively the chemical conditions to which marine calcifiers are exposed. The current and future _xc_[CO_3_^2−^] for the main water masses of the Northeast Atlantic and for the waters where current living communities of CWC exist are reported.

## Results

**Figure 2:**
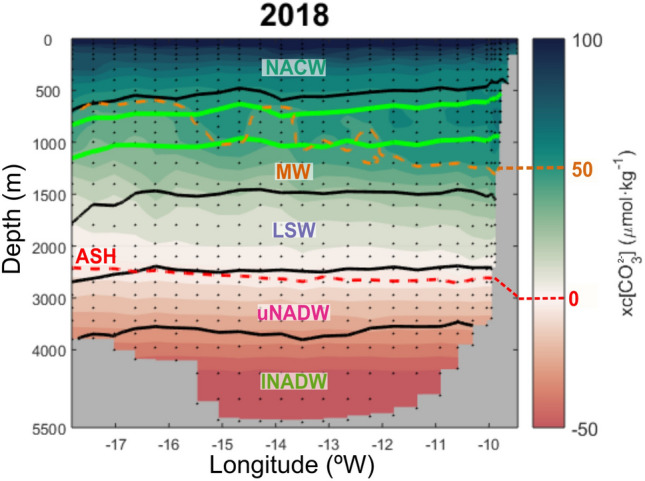
Vertical distribution of excess carbonate with respect to aragonite saturation for the year 2018 at the OVIDE section south of Azores-Biscay Ridge. The plot is the vertical distribution (m) between surface and bottom (maximum depth around 5,500 m at longitude 14° W). The orange dash line is the 50 μmol kg^−1^ isoline and the red dash line is the Aragonite Saturation Horizon (ASH, 0 μmol kg^−1^). The water masses are separated by black lines of potential density according to the layer separation (acronyms and density values are detailed in Table 1). The layer of living cold-water corals (CWC) is represented within the green isopycnals. Note that the depth-scale is not linear.

The vertical distribution of _xc_[CO_3_^2−^] along the OVIDE cruise section for the year 2018 is shown in Fig. 2. The _xc_[CO_3_^2−^] values decrease with depth, from supersaturated (_xc_[CO_3_^2−^] > 0) surface waters with more than 100 μmol kg^−1^ of _xc_[CO_3_^2−^], towards abyssal values of 50 μmol kg^−1^ below saturation (_xc_[CO_3_^2−^] < 0). At mid-depths there are larger _xc_[CO_3_^2−^] values close to the Iberian Peninsula, creating a gradual eastward upward tilt of the isolines. From 3,000 m depth to the bottom, the isolines are horizontal. The isoline of 0 μmol kg^−1^ of _xc_[CO_3_^2−^], known as the Aragonite Saturation Horizon (ASH, red dashed line in Fig. 2), is around 2,500 m depth. With respect to the water masses distribution (Table 1), NACW occupies the top layer down to 700 m at the westward end of the section (that is also the northernmost station, Fig. 1) and to around 500 m close to the Iberian Peninsula. Below, MW extends down to about 1,500 m, encompassing entirely the layer where CWC inhabit between 600 and 1,000 m depth range. Beneath MW, LSW reaches 2,500 m. The lower limit of LSW (σ_2_ = 37.00 kg m^−3^) and the ASH are pretty similar. The limit between the upper and lower NADW is around 3,800 m depth, where waters are already undersaturated (_xc_[CO_3_^2−^] < 0).

**Table 1:** Water mass vertical distribution into layers delimited by potential density (σ_refpressure_) isopycnals.

Water masses	Acronym	Upper limit (kg m^−3^)	Lower limit (kg m^−3^)	References
North Atlantic Central Water	NACW	Surface	σ_0_ = 27.20	^65^
Mediterranean Water	MW	σ_0_ = 27.20	σ_1_ = 32.35	
Labrador Sea Water	LSW	σ_1_ = 32.35	σ_2_ = 37.00	^27^
Upper North Atlantic Deep Water	uNADW	σ_2_ = 37.00	σ_4_ = 45.84	^66^
Lower North Atlantic Deep Water	lNADW	σ_4_ = 45.84	Seafloor	

Water mass names, acronyms, upper and lower limits of potential density and bibliographic references associated.

**Figure 3:**
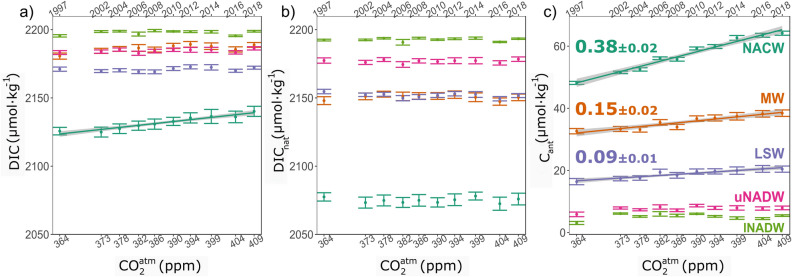
Mean layer concentration trends of dissolved inorganic carbon components in Northeast Atlantic water masses versus atmospheric CO_2_ concentration. Mean layer (**a**) total DIC concentration (μmol kg^−1^) and its separation into (**b**) natural (DIC_nat,_ μmol kg^−1^) and (**c**) anthropogenic (C_ant_, μmol kg^−1^) components versus atmospheric CO_2_ concentration (ppm) for the main water masses in the Northeast Atlantic: NACW (cyan), MW (orange), LSW (purple) and upper and lower NADW (pink and light green). Only linear trends (μmol kg^−1^ ppm^−1^) with a statistical p-value < 0.001 (Supplementary Information Table S1) have been depicted, and the grey shading accounts for the trend errors. Uncertainties in the mean properties are two times the standard error of the mean (i.e., 95% confidence interval). The year of the cruise is represented in the upper x-axis.

The total dissolved inorganic carbon (DIC) mean concentration by water mass (Fig. 3a) shows that the upper layer (NACW) has the lowest values and the bottom layer (lNADW) the largest. In the ocean, the amount of DIC usually increases with depth, but in the Northeast Atlantic the MW influence modifies such vertical distribution. The mid-layer MW has a mean DIC concentration higher than the underlying LSW and very similar to uNADW. The concentration of atmospheric CO_2_ for the period 1997–2018 increased from 364 to 409 ppm (Fig. 3), with an annual mean increase larger than 2 ppm yr^−1^. For DIC, the increase trend with increasing atmospheric CO_2_ concentration is only found in the uppermost water mass, NACW (results of statistical hypothesis test in Supplementary information Table S1).

DIC includes the natural (DIC_nat_) and the anthropogenic (C_ant_) components. DIC_nat_ (Fig. 3b) shows a more monotonous increase from surface to bottom than DIC, although DIC_nat_ concentrations for MW and LSW are very similar. In addition, contrary to the total DIC, DIC_nat_ does not show a trend with increasing atmospheric CO_2_ in any layer. When C_ant_ is analyzed (Fig. 3c), its vertical distribution shows that the surface-most NACW has the highest concentration, which decreases until values slightly above zero in the deeper layers. The increase in C_ant_ with increasing atmospheric CO_2_ is unequivocal in the upper and mid-layers (Supplementary information Table S1), but not for NADW layers. As expected from direct air-sea CO_2_ exchange, the largest increase in C_ant_ occurs in the surface-most NACW layer (0.38 ± 0.02 μmol kg^−1^ ppm^−1^), with concentrations exceeding 60 μmol kg^−1^ by the end of the observational period. MW and LSW mid-layers show rather similar increase in C_ant_ concentration rates (0.15 ± 0.02 μmol kg^−1^ ppm^−1^ and 0.09 ± 0.01 μmol kg^−1^ ppm^−1^, respectively). Note that when time (in years) is used as dependent variable (Fig. S1), the results remain consistent. The vertical distributions of DIC, C_ant_ and DIC_nat_ along the OVIDE cruise section for the year 2018 are shown in Fig. S2.

The vertical distributions of total alkalinity (TA) and TA normalized by salinity (S = 35) along the OVIDE cruise section for the year 2018 are shown in Figure S3. The TA mean concentration by water mass does not show any trend with increasing atmospheric CO_2_ concentration (Fig. S3a). The variation of TA is highly correlated with salinity. The distribution of TA normalized (Fig. S3b) shows a steady increase towards the bottom that is due to CaCO_3_ dissolution but has a very low effect on [CO_3_^2−^]^24^.

**Figure 4:**
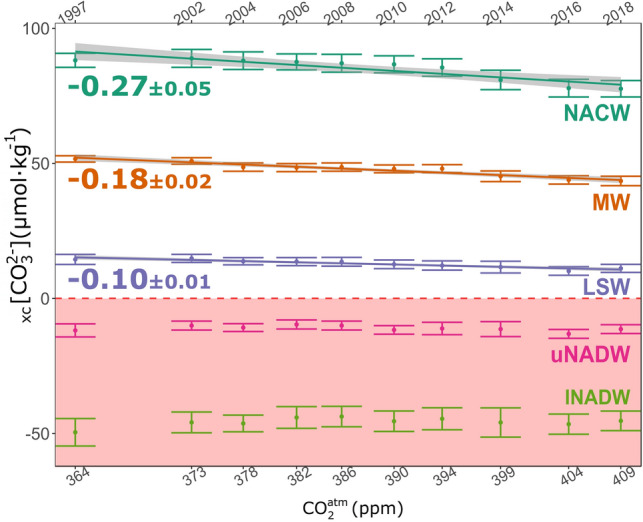
Mean water mass _xc_[CO_3_^2−^] (μmol kg^−1^) versus atmospheric CO_2_ concentration (ppm) in the Northeast Atlantic: NACW (cyan), MW (orange), LSW (purple) and upper and lower NADW (pink and light green). Only linear trends (μmol kg^−1^ ppm^−1^) with a statistical p-value < 0.001 have been depicted (Supplementary Information Table S1), and the grey shading accounts for the trend errors. Uncertainties in the mean properties are two times the standard error of the mean (i.e., 95% confidence interval). The year of the cruise is represented in the upper x-axis. The light red zone below the limit of 0 _xc_[CO_3_^2−^] represents undersaturated waters with respect to aragonite.

The trends of the mean _xc_[CO_3_^2−^] by water mass and associated uncertainties with respect to the atmospheric CO_2_ concentration for 1997–2018 are represented in Fig. 4. The pattern of _xc_[CO_3_^2−^] values between water masses follows a vertical distribution, with positive _xc_[CO_3_^2−^] values at the surface and negative values in deep waters. The _xc_[CO_3_^2−^] decreases with the excess of atmospheric CO_2_ in the upper (NACW, − 0.27 ± 0.05 μmol kg^−1^ ppm^−1^) and mid-layers (MW − 0.18 ± 0.02 μmol kg^−1^ ppm^−1^, and LSW − 0.10 ± 0.01 μmol kg^−1^ ppm^−1^) of the Northeast Atlantic. Such decrease in _xc_[CO_3_^2−^] does not exist in the two layers of NADW (p-values of 0.13 and 0.29 for upper and lower NADW, respectively). The rate of decrease in _xc_[CO_3_^2−^] is almost three times larger at surface than in LSW, with its physicochemical properties and its position in the water column making LSW to be close to the limit of no excess. If these trends persist in time, LSW will become undersatured in the Northeast Atlantic when a concentration of 514 ± 31 ppm of atmospheric CO_2_ is reached.

**Figure 5:**
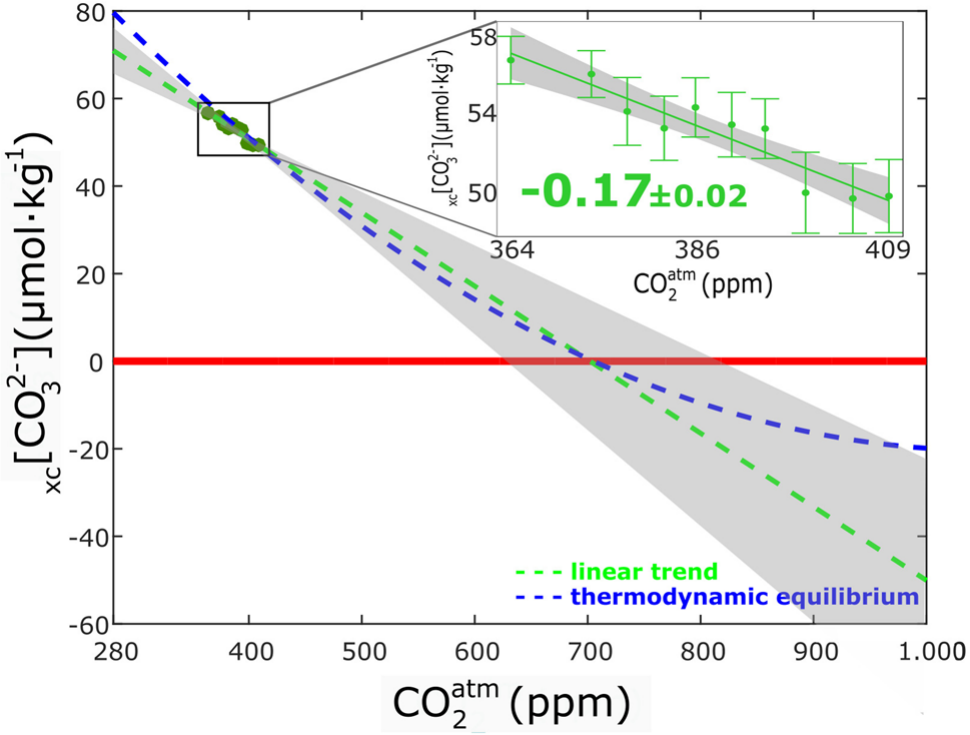
Mean _xc_[CO_3_^2−^] (μmol kg^−1^) versus atmospheric CO_2_ concentration (ppm) for the living cold-water coral (CWC) layer (σ_0_ = 27.35–27.65 kg m^−3^) observed (inset) and predicted. The inset shows the observed measurements and the grey shading accounts for the trend errors. The linear trend in the inset (green line, μmol kg^−1^ ppm^−1^) has a statistical p-value = 4.3 × 10^–5^. In the inset, the uncertainties in the mean properties are two times the standard error of the mean (i.e., 95% confidence interval), and the year of the cruise is represented in the upper x-axis. The predictions (dashed lines) are based on the linear rate of decrease for the observational period (green) and on the thermodynamic equilibrium trend (blue, Orr et al.^10^).

In the depth range of living CWC, the change in _xc_[CO_3_^2−^] with increasing atmospheric CO_2_ shows a decrease from 56 to less than 50 μmol kg^−1^ during the observing time frame (inset in Fig. 5). The rate of decrease is − 0.17 ± 0.02 μmol kg^−1^ ppm^−1^ (p-value = 4.3 × 10^–5^). Conserving this linear trend, the current layer of living CWC in the Northeast Atlantic would then be in undersaturated waters at a concentration of 702 ± 53 ppm of atmospheric CO_2_ (Fig. 5). The nonlinearity between ocean carbon variables requires a thermodynamical approach to infer distant projections^10^. However, within a thermodynamic equilibrium, the layer of living CWC approximately follows the linear trend (Fig. 5). The large loss of linearity occurs below the undersaturation level (_xc_[CO_3_^2−^] < 0), so the linear trend is a valid approach for the study purposes.

## Discussion

In the huge pool of inorganic carbon existing in the ocean, the anthropogenic component represents a relatively small fraction of it (< 4%)^2^. Current anthropogenic perturbation in NACW layer (< 600 m depth, approximately) is large enough to be detected in the DIC pool within a 21-year timespan (1997–2018) (Fig. 3a). The detection of trends in DIC, however, may require longer time series if the measurements are not normalized by salinity^6^ or alkalinity. In the Northeast Atlantic, the increase in DIC is not mediated by a change in total alkalinity (Fig. S3). And even without salinity normalization, the large anthropogenic perturbation, combined with the fact that all measurements were made in the same season (summer), favors the detection of trends^5^. This 21-year time-scale agrees well with the 14-year period required to detect the emergence of the anthropogenic signal from natural variability in another carbon variable, the surface pH^25^. In contrast, the natural DIC shows no change at any layer (Fig. 3b, Supplementary Information Table S1) suggesting that, within this approach, we cannot discard the steady-state of the natural carbon cycle in the Northeast Atlantic. Nevertheless, the capability of current anthropogenic carbon methods to discern changes in the natural DIC is still a debated subject^26^ that is out of the scope of the present study. For the C_ant_, increasing trends are unequivocally observed not only in the upper NACW layer but also in the mid-layer water masses, i.e., until 2,500 m depth. There is a consistent increase in C_ant_ in the upper and mid-layers of the Northeast Atlantic that responds to the atmospheric CO_2_ increase over time. These results agree, and update by more than a decade, the previously reported C_ant_ trends in the Northeast Atlantic^27^. The C_ant_ increase in the NACW layer is among the largest trends reported in the literature^9,27^, in agreement with the view that the subtropical areas are the places where the upper water masses increase its C_ant_ burden^3,28–30^. South of the study zone, in the Gulf of Cadiz region (Fig. 2), the downwelling of central waters with high C_ant_ concentrations and the subsequent formation of MW exports C_ant_ to the Northeast Atlantic^31^. This convection of the anthropic signal at mid-depths is then advected, spreading acidified waters to the North Atlantic. Although the anthropogenic influence has already reached the old water masses (NADW) of the Iberian Abyssal Plain, a longer monitoring period is required to identify it. In summary, the combination of the natural component of DIC in steady-state with the unequivocal trends for C_ant_ in NACW, MW and LSW, makes it reasonable to assume that the existence of perturbations in other marine carbonate system variables are also produced by human influence, as seen for pH, CO_3_ (Fig. S4, Table S1) and _xc_[CO_3_^2−^] (Fig. 4). The latter is the variable that we are going to consider hereafter.

_xc_[CO_3_^2−^] decreases with depth due to solubility (high pressure and low temperature increases aragonite solubility). The anthropogenic decline of _xc_[CO_3_^2−^] exists in all the supersaturated water masses, that is, NACW, MW and LSW. The decline is happening three times faster in NACW than in LSW, as expected from the atmospheric source of the driver for ocean acidification (atmospheric CO_2_) and the age of these water masses^9^. The relatively young and recently formed NACW has the largest trend, whereas LSW, ventilated in western subpolar latitudes, reaches the Northeast Atlantic basin with a greater mean age and hence has the lowest trend among the acidified water masses. MW, formed at the southeast of the Iberian Peninsula (Gulf of Cadiz, Fig. 2) by mixing of NACW, Mediterranean Outflow Water (MOW) and a diluted form of Antarctic Intermediate Water^32,33^, shows intermediate trends. At the latitudes of the measurements, the layer with living CWC is comprised within the MW layer, between 600 and 1,000 m depth. Therefore, the living CWC are in waters with excess of available carbonate, in agreement with the idea that suitable habitats for CWC development are supersaturated for aragonite^18^. MOW contributes for around a third (~ 34%) in the composition of MW^32^. Due its intrinsic properties of high salinity and alkalinity^34,35^, MW is therefore crucial to keep the Northeast Atlantic CWC in chemically optimal waters. In the Gulf of Cadiz, the ocean acidification rates in MW are relatively high^36^. Currently, living CWC are located between the fast-acidifying surface waters from above and the rising ASH from below. A potential reduction in MOW by global change^37^ would involve a greater influence of subpolar origin waters that could accelerate the exhaustion of excess carbonate in the Northeast Atlantic.

**Figure 6:**
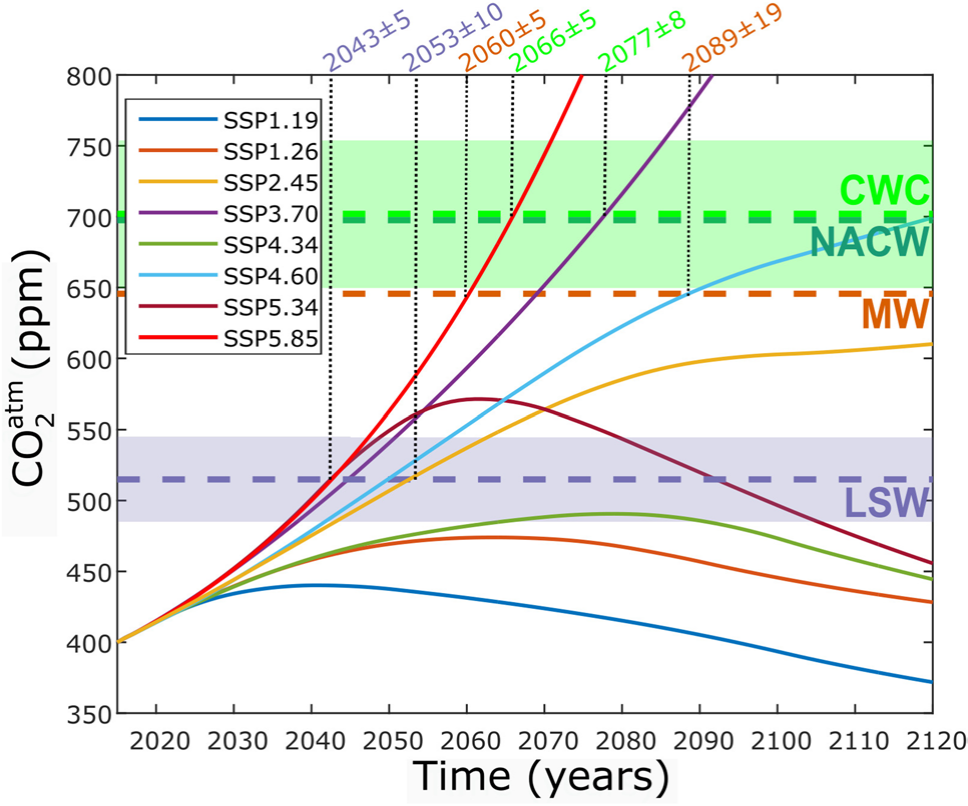
Projections of atmospheric CO_2_ concentration (ppm) versus time (years) as modelled by the eight Shared Socioeconomic pathways (SSPs) considered. Atmospheric CO_2_ data from^39^. The horizontal dashed lines are the predicted atmospheric concentration at the moment of undersaturation for each layer. The predicted uncertainty for the LSW and CWC layers is the 95% confidence interval at the moment of undersaturation for the projected mean properties linear trends; and it is represented with the light-colored horizontal band. For specific pathways, the expected year of reaching undersaturation along with their uncertainty is included in the upper axis.

Reaching undersaturation for aragonite in the upper and mid-layers of the Northeast Atlantic would take less than a century in worst scenarios (SSP5.85, SSP4.60 and SSP3.70, Fig. 6). Fossil-fueled development pathway SSP5.85 is similar to that projected by the previous IPCC business-as-usual scenario (RCP8.5)^38^. The scenarios that best represent the limitation of global warming to 1.5 or 2 °C of the Paris Agreement are the SSP1-19 and the SSP1-26 respectively^39^. For both scenarios, the decline of _xc_[CO_3_^2−^] will not reach undersaturation values not even in LSW layer (Fig. 6). Therefore, if the CO_2_ emission targets for limiting global warming to 1.5 or 2 ℃ are accomplished, the Northeast Atlantic will remain in chemically optimal conditions for CWC communities the following century. However, if the atmospheric CO_2_ concentration reached 700 ppm, then the living CWC communities in the Northeast Atlantic would be exposed to waters that are chemically hostile to their carbonate structures. Our projection based in measurements is in agreement with previous model forecasts^10,18^.

Many species of benthic calcifiers, even CWC other than *Lophelia pertusa*, inhabit undersaturated seawaters^40,41^. Although living CWC can have net calcification in undersaturated conditions^42,43^, they do it at expenses of stored energy reserves^44^. Three-dimensional CWC reefs structures are composed by an important fraction (> 70%) of dead corals framework^45^. In contrast to the living CWC, the dissolution of the dead skeletons is a predicted chemical reaction^46^ because the skeletons have no capacity to cope with dissolution. The net dissolution will be directly proportional to the time CWC remain in undersaturated waters and that could happen within a century if the Paris Agreement is not accomplished. To the best of our knowledge, there are no long-term in situ experiments that confirm net dissolution of dead CWC framework available at this time. However, the combination of bioerosion proceeding faster in substrates weakened by ocean acidification^47^ with the close relationship between carbonate availability and the location of healthy CWC communities^4,18,19^ suggests that CWC development might be compromised. Furthermore, the decrease in available carbonate saturation level may combine with, and potentially exacerbated by, other climate change pressures such as warming and deoxygenation^48,49^.

## Conclusions

The uptake of anthropogenic carbon from the atmosphere is unequivocally decreasing the amount of carbonate available for marine calcifiers in the upper (NACW) and intermediate water masses (MW and LSW) of the Northeast Atlantic Ocean. The northward spreading of MW plays a key role in limiting the arrival of subpolar-origin waters with low excess of carbonate available. The chemical conditions that made the Northeast Atlantic a region favorable for CWC development in preindustrial time are changing fast due to ocean acidification. Currently, provided the water masses contribution remain the same, living CWC would inhabit undersaturated waters when the atmospheric CO_2_ concentration reaches 702 ± 57 ppm. If greenhouse gas emissions maintain the path of fossil-fuel development, the running out of excess carbonate in the Northeast Atlantic will take place during this century. The efficiency of high seas Marine Protected Areas (MPA) created for the long-term conservation of CWC and their associated ecosystems is ultimately associated with the accomplishment of the Paris Agreement and the limitation of global warming below 2 ℃.

## Materials and methods

Two-decades of ocean acidification trends across the whole water column of the Northeast Atlantic are evaluated by means of high-quality CO_2_ measurements. The observations came from ten hydrographic cruises across the Iberian Abyssal Plain between 1997 and 2018 (Fig. 2): the 1997 FOUREX cruise (CLIVAR Carbon Hydrographic Data Office site, https://cchdo.ucsd.edu/cruise/74DI230_1) and 9 repeats of the OVIDE section (OVIDE group, 2020). They are almost evenly spaced in time since all cruises except the first one belong to the OVIDE sampling program, a high-resolution hydrographic survey that has been carried out every other year during spring–summer since 2002 (https://www.umr-lops.fr/en/Projets/Projets-actifs/OVIDE). The assembled high-quality CO_2_ system database between the Azores Biscay Ridge (45° N, 18° W) and the Iberian Peninsula therefore spans 21 years (1997–2018; Fig. 2) and the whole-water column (Fig. 1). Note the cruise track is identical for the nine repetitions of the OVIDE section, but it is slightly southern for the 1997 cruise. Both coast-to-coast hydrographic sections belong to GO-SHIP (Global Ocean Ship-Based Hydrographic Investigations Program), which is part of the global ocean/climate observing systems (GOOS/GCOS)^50^. Note here we are analyzing their easternmost part (< 18° W) exclusively.

### Measured carbon variables: pH and total alkalinity

In this study, the carbon analysis for all the hydrographic data involved followed the same analytical methodology and were in situ calibrated against Certified Reference Materials (CRMs).

pH was determined with a spectrophotometric method^51^. The protocols of measurements developed and followed during the cruises, including periodical CRM checks, allowed to achieve an internal consistency and reproducibility of ± 0.0014 pH units^9,27^. The large amount of measurements from the deeper waters sampled at the Iberian Abyssal Plain (n = 1,633) show a very low standard deviation (7.9146 ± 0.0006 pH at in situ conditions of temperature and pressure in the total scale), which is a useful indicator of consistency and reproducibility since these old waters are expected to be in near steady state^9^. Total alkalinity (TA) was analyzed by single point titration^52^ and calibrated with CRM with a measurement precision of ± 2 μmol kg^−1^. Over 3,200 samples of TA were analyzed for this study, with around three hundred per cruise for the stations shown in Fig. 2. The amount of total alkalinity measurements with WOCE flags values different from “Acceptable” is less than 1%. The datasets were subject to primary and secondary quality control procedures^53^ consistent with the GLODAP data products^54,55^ and neither carbon related variables (pH and total alkalinity) nor oxygen have been modified at the secondary quality control procedure^55^. Then, results are supported by high-quality and low-uncertainty carbon measurements spanning 21 years.

### Computed carbon variables

Concentrations of dissolved inorganic carbon (DIC) and in situ carbonate ions ([CO_3_^2−^]_is_) were calculated with the CO2SYS toolbox^56^ using the acid dissociation constants of Mehrbach et al*.*^57^ refitted by Dickson and Millero^58^, and the aragonite solubility of Mucci^59^. The uncertainty in the concentrations is ± 4.6 μmol kg^−1^ and ± 3.7 μmol kg^−1^ for DIC and [CO_3_^2−^]_is_, respectively^60^.

The in situ degree of aragonite saturation (Ω_Arg_) is the product of the ion concentrations of calcium ([Ca^2+^]) and carbonate ([CO_3_^2−^]) divided by the aragonite solubility product (K_Arg_) at in situ conditions (subscript “_is_”) of temperature, salinity, and pressure^12^. Following the Ω_Arg_ definition:

1 $${\Omega }_{\mathrm{Arg}}= \frac{\left[{\mathrm{Ca}}^{2+}\right]{\left[ {\mathrm{CO}}_{3}^{2-}\right]}_{\mathrm{is}}}{{\mathrm{K}}_{\mathrm{Arg}}},$$

we can infer [CO_3_^2−^] at saturation (i.e., when Ω_Arg_ equals one):

2 $${\left[{\mathrm{CO}}_{3}^{2-}\right]}_{\mathrm{sat}\left({\Omega }_{\mathrm{Arg}}=1\right)}= \frac{{\mathrm{K}}_{\mathrm{Arg}}}{\left[{\mathrm{Ca}}^{2+}\right]},$$

where the concentration of the conservative ion [Ca^2+^] is determined only by salinity^61^. Both K_Arg_ and [Ca^2+^] were calculated with the CO2SYS toolbox^56^. The difference between [CO_3_^2−^]_is_ and [CO_3_^2−^]_sat(ΩArg = 1)_ (Eq. 2) is the excess of carbonate ion concentration over aragonite saturation: _xc_[CO_3_^2−^]^4,11^. Note that an alternative estimate is the _xc_[CO_3_^2−^] over calcite saturation, computed with the degree of calcite saturation (Ω_Calcite_). Since calcite is more resistant to dissolution than aragonite^11^, calcifying organisms that form their structures with calcite will be less affected by ocean acidification at shorter time-scales^62^. Therefore, in this work, we refer exclusively to excess carbonate and the saturation state with respect to aragonite.

Finally, anthropogenic carbon (C_ant_) was estimated with the biogeochemical back-calculation ϕC_T_° method^63^, which has an overall uncertainty of ± 5.2 μmol kg^−1^. The natural fraction in the total DIC (DIC_nat_) is the difference between DIC and C_ant_.

### Layer separation

The whole-water column was separated following a vertical water mass distribution into five layers delimited by potential density (σ_refpressure_) isopycnals (Table 1, Fig. 1), following previous studies in the area^8,27^. The reference pressure level for the isopycnals (refpressure) varies among 0, 1, 2 and 4 (× 10^3^ dbar).

In addition, we also delimited the range of water column where living CWC exist in the Northeast Atlantic^20^, i.e. 27.35 ≤ σ_0_ ≤ 27.65 kg m^−3^ (Fig. 1).

For each layer, the mean property concentrations (_xc_[CO_3_^2−^], DIC, DIC_nat,_ C_ant_, TA pH_is_T and [CO_3_^2−^]_is_) were based on interpolated bottle data at dbar resolution. Interpolation was done on an area-weighted basis, considering the thickness of the layer and the distance between measurements^9^. The mean properties are represented along with the 95% confidence interval (error bars are two times the standard error of the mean, *2x(std/√n)*; where *n* is the number of bottle measurements in that layer and cruise, Figs. 3, 4, 5).

### Atmospheric CO_2_: past concentrations and shared socioeconomic pathways projections

In this study we contrasted the anthropogenic changes in the ocean carbon cycle with respect to its main driver, the atmospheric CO_2_ concentration, in contrast to previous studies in the region using time as dependent variable^8,9^. Note that this methodology develops rates with respect to atmospheric CO_2_ rather than time, so the units of the rates are μmol kg^−1^ ppm^−1^. Atmospheric CO_2_ concentration is a more meaningful variable than time, since it allows to make projections directly based on greenhouse gas concentrations resulting from different policy decisions and socioeconomic pathways. Past atmospheric CO_2_ concentrations were taken from the Mauna Loa database (https://www.esrl.noaa.gov/gmd/ccgg/trends/) [last time accessed: 28/12/2019]. In this study we used the mean annual concentration of CO_2_ (in parts per million of volume, p.p.m.) for the year of the cruise. Shared Socioeconomic Pathways (SSP) are climate scenario frameworks that take into account a wide range of socio-economic futures and policy decisions^38^. Atmospheric CO_2_ concentrations for eight representative SSP scenarios belonging to the Coupled Model Intercomparison Project phase 6 (CMIP6) were downloaded from https://greenhousegases.science.unimelb.edu.au [last time accessed: 28/12/2019]^39^. This data is used in the atmospheric CO_2_ concentration projections.

## Supplementary information

Supplementary Information 1.

## Data Availability

Data were collected and made publicly available by the International Global Ship-based Hydrographic Investigations Program (GO-SHIP; https://www.go-ship.org/) and the national programs that contribute to it. Global Distribution of Cold-water Corals version 5.0 (June 2018) is distributed under UNEP-WCMC's General Data License (excluding WDPA). URL: https://data.unep-wcmc.org/datasets/3.
