# Supplementary material for: The Northeast Atlantic is running out of excess carbonate in the horizon of cold-water corals communities
Source: Sci Rep. 2020 Sep 7;10:14714. doi: 10.1038/s41598-020-71793-2 (PMC7477189; doi:10.1038/s41598-020-71793-2)
Supplement: Supplementary file 1 — Supplementary Information 1. [file 41598_2020_71793_MOESM1_ESM.pdf]

Supplementary Information of:

The Northeast Atlantic is running out of excess carbonate in the horizon of cold-water corals communities

Marcos Fontela[1,2*], Fiz F. Pérez[1], Lidia I. Carracedo[3], Xosé A. Padín[1], Antón Velo[1], Maribel I. García-Ibañez[1,4], Pascale Lherminier[3]

[1] Instituto de Investigaciones Marinas, IIM-CSIC, 36208 Vigo, Spain.

[2] Center of Marine Sciences (CCMAR), Universidade do Algarve, 8005-139 Faro, Portugal.

[3] Ifremer, Univ. Brest, CNRS, IRD, Laboratoire d'Océanographie Physique et Spatiale (LOPS), IUEM, F-29280, Plouzané, France
[4] Centre for Ocean and Atmospheric Sciences, School of Environmental Sciences, University of East Anglia, Norwich, UK

Corresponding author: Marcos Fontela (mmfontela@ualg.pt)

Supplementary Table S1. Statistical results of linear trends (p-values). P-value lower than 0.001 are highlighted in bold.

VS CO_2 (ppm)	DIC	C_{ant}	DIC_{nat}	NTA	xc[CO_3^{2-}]	[CO_3] $_{is}$	$pH_{is}T$
**NACW**	**4.9E-06**	**8.4E-08**	0.87	0.63	**5.7E-04**	**7.6E-04**	**3.4E-05**
**MW**	0.01	**2.7E-05**	0.81	0.39	**1.24E-05**	**3.0E-05**	**3.8E-06**
**LSW**	0.23	**3.6E-05**	0.16	0.15	**5.50E-05**	**3.4E-05**	**1.8E-04**
**uNADW**	0.14	0.09	0.74	0.55	0.23	0.13	0.05
**lNADW**	0.62	0.60	0.83	0.44	0.23	0.29	0.37
**CWC**	**6.6E-04**	**6.2E-05**	0.31	0.25	**4.3E-05**	**9.4E-05**	**2.9E-06**

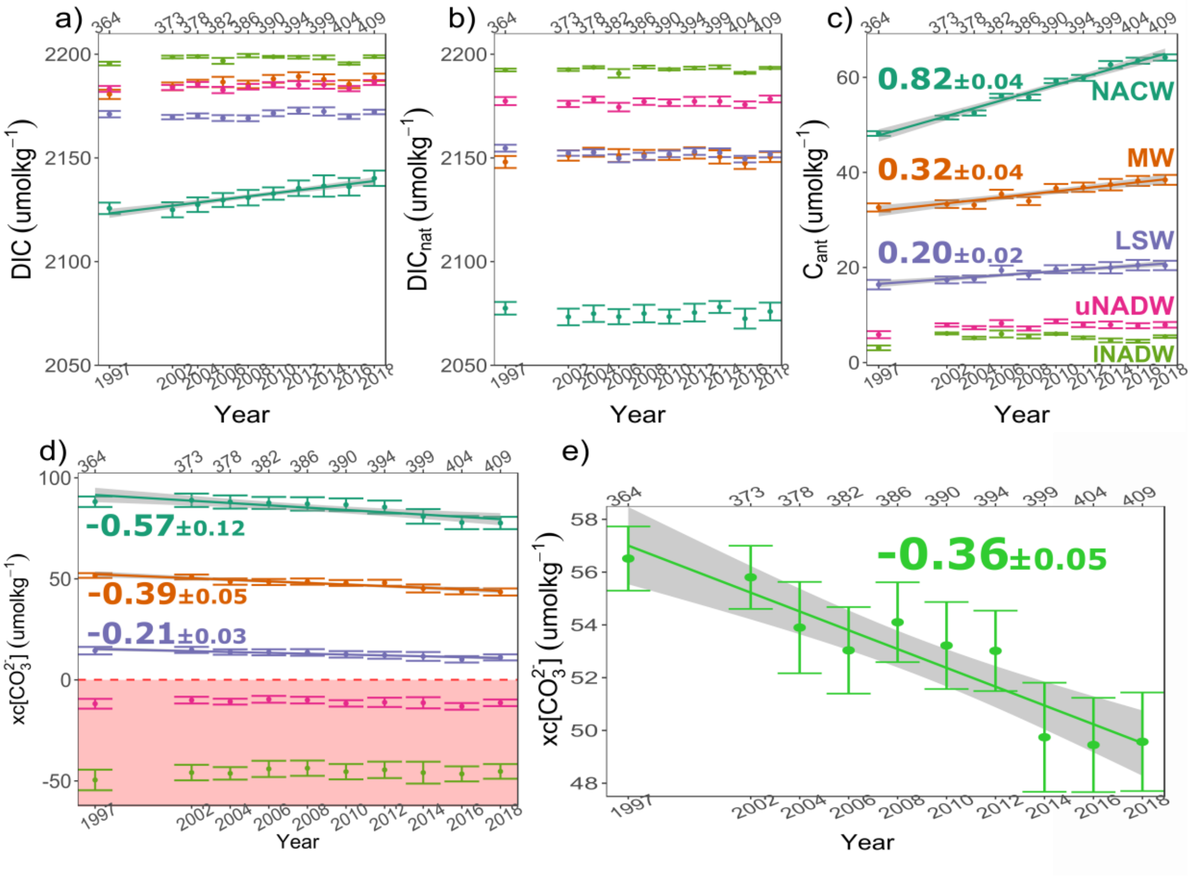

Supplementary Figure S1. Mean layer concentration trends of carbon components versus time in Northeast Atlantic water masses. Mean layer a) total DIC concentration (µmol kg^{-1}), b) natural DIC (µmol kg^{-1}), c) anthropogenic DIC (µmol kg^{-1}) and d) $_{xc}$[CO_3^{2-}] (µmol kg^{-1}) versus time (year) for the main water masses in the Northeast Atlantic: NACW (cyan), MW (orange), LSW (purple), and upper and lower NADW (pink and light green). e) Mean $_{xc}$[CO_3^{2-}] (µmol kg^{-1}) versus time (year) for the living cold-water coral (CWC) layer (σ_0=27.35-27.65 kg m^{-3}). Only linear trends (µmol kg^{-1} yr^{-1}) with a statistical p-value <0.001 (Supplementary Information Table S1) have been depicted, and the grey shading accounts for the trend errors. Uncertainties in the mean properties are two times the standard error of the mean (i.e., 95% confidence interval). In d), the light red zone below $_{xc}$[CO_3^{2-}]=0 represents undersaturated waters with respect to aragonite The annual atmospheric CO_2 concentration (ppm) is represented in the upper x-axis.

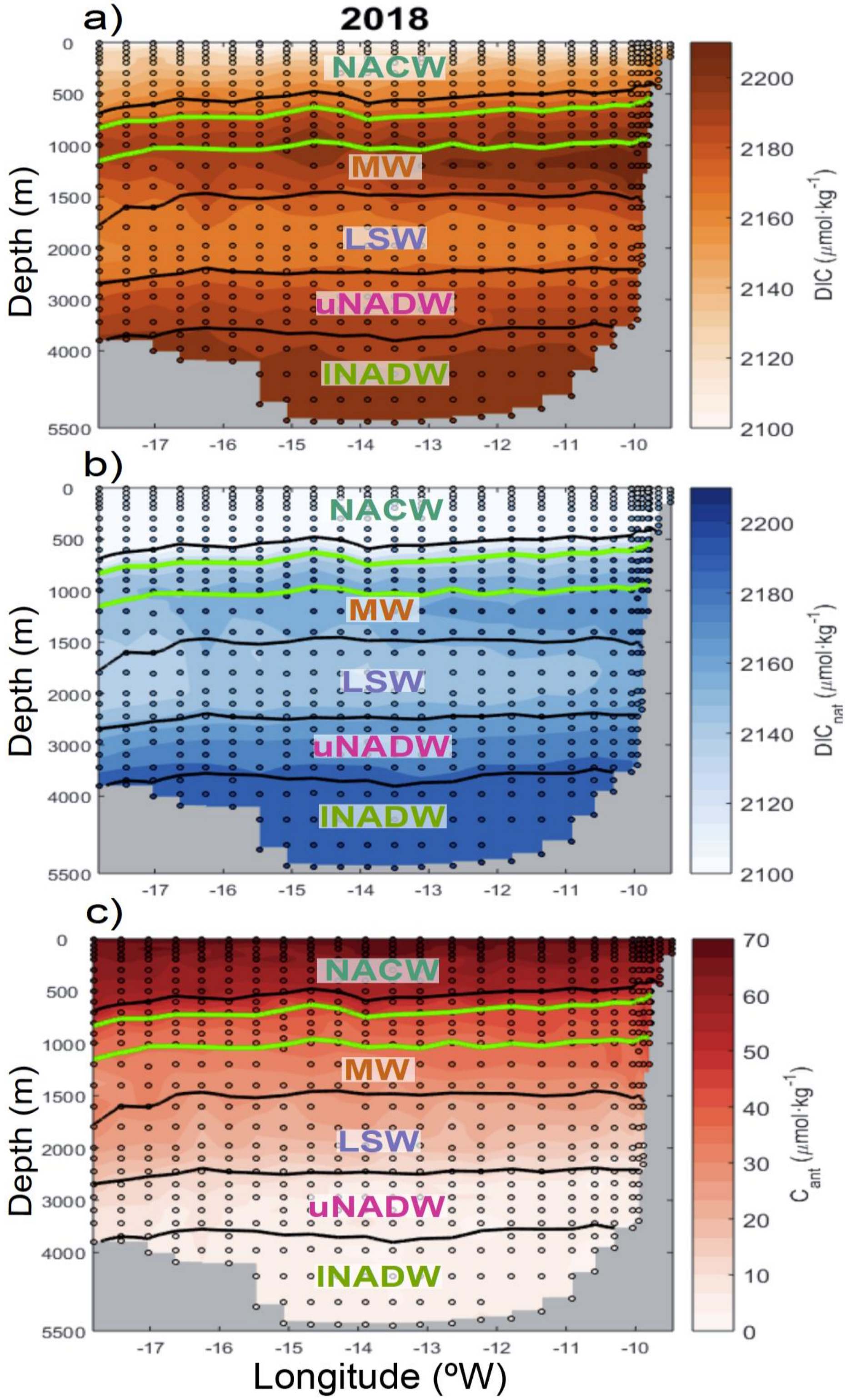

Supplementary Figure S2. Vertical distribution of carbon components. Vertical distribution of a) DIC, b) DIC_{nat} and c) C_{ant} for the year 2018 at the OVIDE section south of Azores-Biscay Ridge (µmol kg^{-1}). The plot is the vertical distribution (m) between surface and bottom (maximum depth around 5500 m at longitude 14ºW). The water masses are separated by black lines of potential density according to the layer separation (Table 1). The layer of living CWC is represented within the green isopycnals. Note that the depth-scale is not linear.

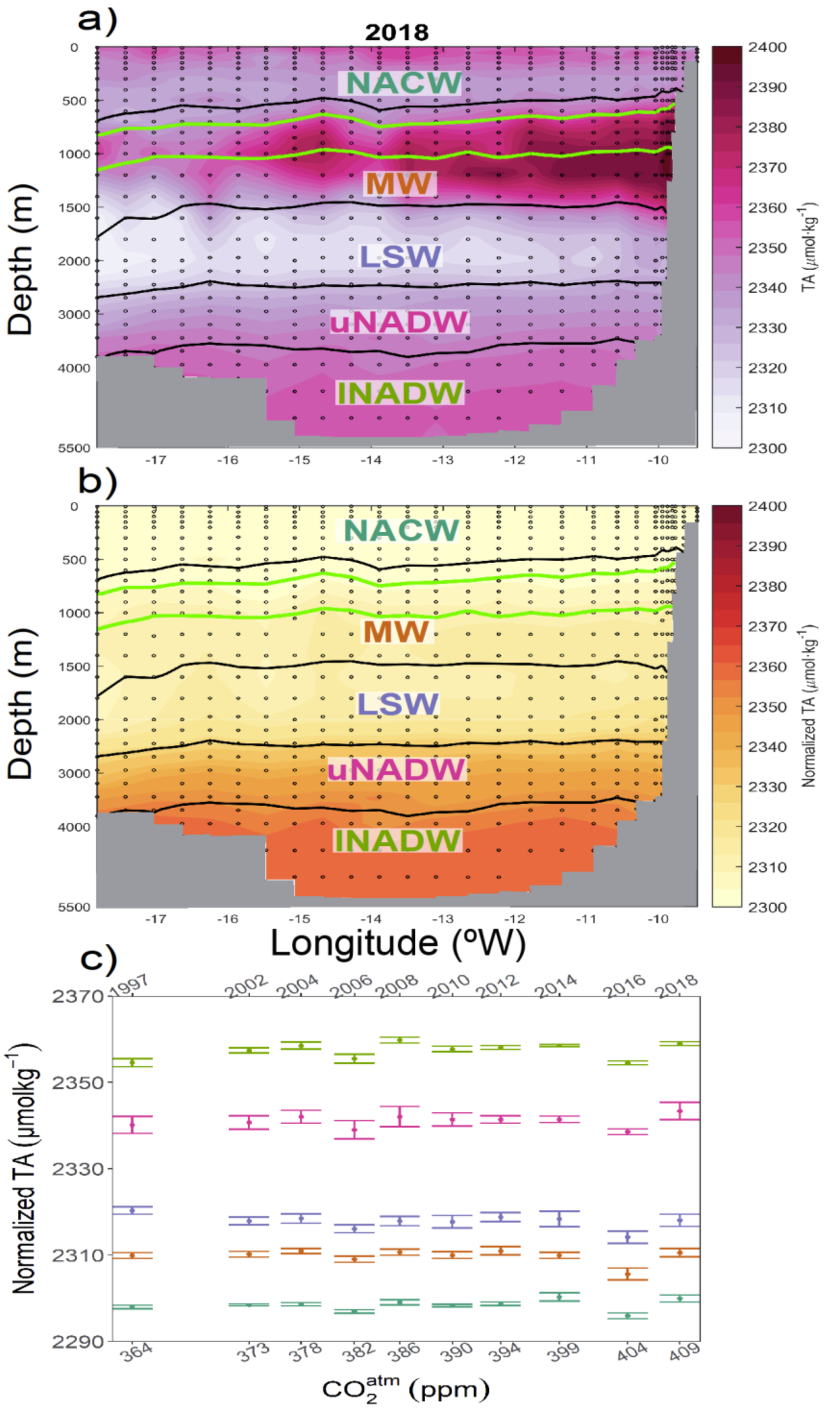

Supplementary Figure S3. Alkalinity data. a) Vertical distribution of Total alkalinity (TA), and b) TA normalized by salinity (S=35, Normalized TA) for the year 2018 at the OVIDE section south of Azores-Biscay Ridge (μmol kg^{-1}). The plot is the vertical distribution (m) between surface and bottom (maximum depth around 5500 m at longitude 14ºW). The water masses are separated by black lines of potential density according to the layer separation (Table 1). The layer of living CWC is represented within the green isopycnals. Note that the depth-scale is not linear. c) Mean layer concentration trends of normalized TA (μmol kg^{-1}) versus atmospheric CO_2 concentration for the main water masses in the Northeast Atlantic: NACW (cyan), MW (orange), LSW (purple) and upper and lower NADW (pink and light green). Uncertainties in the mean properties are two times the standard error of the mean (i.e., 95% confidence interval). The year of the cruise is represented in the upper x-axis.

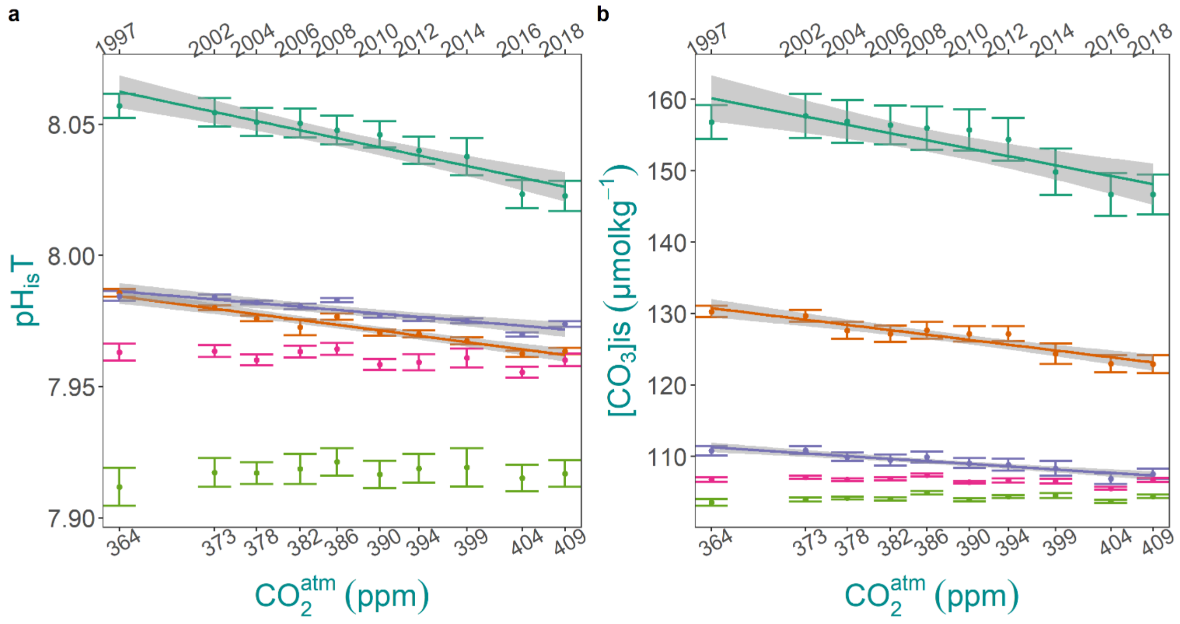

Supplementary Figure S4. Mean layer concentration trends of pH and *in situ* carbonate versus atmospheric CO_2 concentration in Northeast Atlantic water masses. a) *in situ* pH at total scale and b) mean *in situ* $[CO_3^{2-}]$ concentration (µmol kg^{-1}) versus atmospheric CO_2 concentration (ppm) for the main water masses in the Northeast Atlantic: NACW (cyan), MW (orange), LSW (purple) and upper and lower NADW (pink and light green). Only linear trends (µmol kg^{-1} ppm^{-1}) with a statistical p-value <0.001 (Supplementary Information Table S1) have been depicted, and the grey shading accounts for the trend errors. The year of the cruise is represented in the upper x-axis.
